# Role of GDF15 in active lifestyle induced metabolic adaptations and acute exercise response in mice

**DOI:** 10.1038/s41598-019-56922-w

**Published:** 2019-12-27

**Authors:** Carla Igual Gil, Mario Ost, Juliane Kasch, Sara Schumann, Sarah Heider, Susanne Klaus

**Affiliations:** 1 Department of Physiology of Energy Metabolism, German Institute of Human Nutrition Potsdam-Rehbrücke, Nuthetal, Germany; 2 University of Potsdam, Institute of Nutritional Science, Potsdam, Germany

**Keywords:** Homeostasis, Obesity, Obesity

## Abstract

Physical activity is an important contributor to muscle adaptation and metabolic health. Growth differentiation factor 15 (GDF15) is established as cellular and nutritional stress-induced cytokine but its physiological role in response to active lifestyle or acute exercise is unknown. Here, we investigated the metabolic phenotype and circulating GDF15 levels in lean and obese male C57Bl/6J mice with long-term voluntary wheel running (VWR) intervention. Additionally, treadmill running capacity and exercise-induced muscle gene expression was examined in GDF15-ablated mice. Active lifestyle mimic via VWR improved treadmill running performance and, in obese mice, also metabolic phenotype. The post-exercise induction of skeletal muscle transcriptional stress markers was reduced by VWR. Skeletal muscle GDF15 gene expression was very low and only transiently increased post-exercise in sedentary but not in active mice. Plasma GDF15 levels were only marginally affected by chronic or acute exercise. In obese mice, VWR reduced GDF15 gene expression in different tissues but did not reverse elevated plasma GDF15. Genetic ablation of GDF15 had no effect on exercise performance but augmented the post exercise expression of transcriptional exercise stress markers (*Atf3*, *Atf6*, and *Xbp1s*) in skeletal muscle. We conclude that skeletal muscle does not contribute to circulating GDF15 in mice, but muscle GDF15 might play a protective role in the exercise stress response.

## Introduction

The development of metabolic diseases is affected not only by dietary factors and energy intake but also by physical activity as an important determinant of energy expenditure. In humans exercise improves cardiorespiratory fitness and metabolic biomarkers^1^. An active lifestyle improves health in general by counteracting obesity associated metabolic dysfunctions^2,3^.

Skeletal muscle is a highly dynamic organ subjective to adaptive remodelling in response to aging, starvation, metabolic disorders, and, importantly, physical exercise^4^. An acute bout of strenuous exercise imposes a severe physiological stress resulting in the activation of specific signaling pathways regulating exercise-induced gene expression and protein synthesis^5^. Skeletal muscle adaptations to exercise training are the result of repeated, transient bursts in mRNA expression during each exercise bout that have a cumulative effect and lead to increases in transcription of nuclear- and mitochondrial-encoded proteins^6^. Strenuous exercise can lead to skeletal muscle injury causing an induction of endoplasmic reticulum (ER) stress and the unfolded protein response (UPR) pathways which play a pivotal role leading to either improvement or deterioration of skeletal muscle mass and function. In mice, it has been shown that the UPR mediates skeletal muscle adaptation to exercise^7^ and repeated activation of the UPR by exercise is an important contributor to exercise induced skeletal muscle remodelling^8^. Moreover, it is well accepted that skeletal muscle is a secretory organ that releases so-called myokines, i.e. muscle-derived secretory proteins, into the circulation to act on different organs in an auto/paracrine or endocrine manner^9^. A recent study suggested a potential role of growth differentiation factor 15 (GDF15) during exercise in humans where increasing plasma concentrations were found both during and after exercise in young, healthy adults^10^. In elite rugby players, acute exercise increased circulating GDF15 levels and they correlated with cardiovascular risk^11^. Additionally, in old obese adults, GDF15 has been reported to have a role in fat mass reduction during exercise training^12^. GDF15 was originally identified as a member of the transforming growth factor β superfamily but recently reclassified as a member of the Glial cell-derived neurotropic factor (GDNF) family which binds to GDNF family receptor α-like (GFRAL) protein, a transmembrane receptor exclusively expressed in the hind brain^13^. GDF15 circulating levels are normally very low but can be substantially increased in various diseases including cancer and metabolic diseases^13,14^. GDF15 is known to be induced by various cellular stresses or stimuli in a variety of tissues and cell types, and therefore regarded as a biomarker for various pathologies including diabetes and cardiovascular disease^15,16^, mitochondrial disease^17^, and obesity^18^.

Here we addressed the possible role of GDF15 in exercise performance, acute exercise response, and skeletal muscle exercise adaptation in mice. For that, we made use of voluntary wheel running (VWR) as an active lifestyle mimic in lean and obese mice. VWR enhances exercise capacity in mice^19,20^ and prolongs health-span by preventing the age-related decline of aerobic fitness and motor coordination^21^. Here we investigated the acute skeletal muscle transcriptional response to an exhaustive exercise bout in sedentary and active mice, and the effect of VWR on circulating GDF15. Furthermore, exercise capacity and post exercise muscle gene expression were examined in GDF15-ablated mice.

## Results

### An active lifestyle induces skeletal muscle exercise adaptation but does not affect circulating GDF15

**Figure 1:**
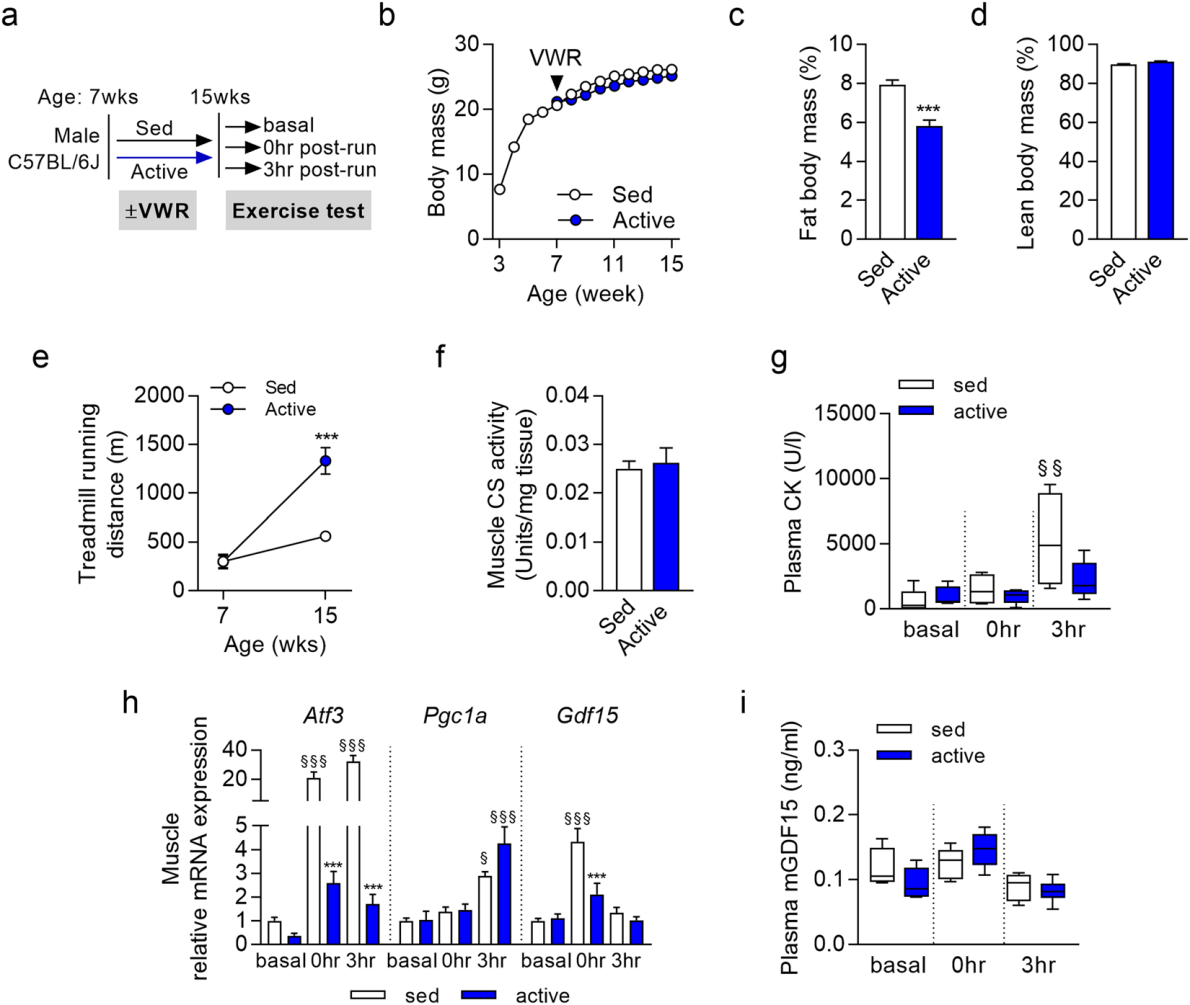
Circulating GDF15 is not affected by acute treadmill exercise or long-term voluntary wheel running (VWR) in mice. (**a**) Study setup: Male mice were fed a semisynthetic low fat diet throughout life. Half of the mice were provided a running wheel from 7 wks of age (active group). All mice were subjected to an exhaustive treadmill test at 15 weeks of age and groups of mice sacrificed before (basal), immediately (0 hr post-run), and 3 hours (3 hr post-run) after the exercise bout, respectively; (**b**) Body weight development (n = 22–65 per group); (**c**) Body fat mass (n = 30–35) at 15 weeks of age; (**d**) Body lean mass (n = 30–35) at 15 weeks of age; (**e**) Development of exercise capacity determined by forced treadmill exercise until exhaustion (n = 9–10 per group); (**f**) Muscle citrate synthase (CS) activity (n = 12 per group); (**g**) Plasma creatine kinase (CK) (n = 4–7 per group); (**h**) Skeletal muscle (quadriceps) gene expression of *Atf3*, *Pgc1α* and *Gdf15* (n = 5–6 per group); (**i**) Plasma GDF15 concentrations (n = 5–6 per group). Data are presented as mean + SEM (**b**–**f**,**h**) or as box plot with whiskers indicating minimum and maximum values (**g**,**i**); *p < 0.05, **p < 0.01, ***p < 0.001 compared to the respective sedentary group. ^§^p < 0.05; ^§§^p < 0.01; ^§§§^p < 0.001 compared to basal control of the same activity group.

In order to assess the potential role of GDF15 during exercise adaptation, we provided wild-type (WT) C57BL/6J mice at 7 wks of age with a running wheel (active) and compared them with mice provided with no running wheel (sedentary). At 15 wks of age sedentary and active mice were sacrificed at basal state, immediately after an acute exhaustive exercise bout (0 hr post-run), and 3 hours after the exhaustive exercise bout during the recovery phase (3 hr post-run), respectively (Fig. 1a). Body weight development was not affected by VWR (Fig. 1b) besides a slight reduction in body fat mass (Fig. 1c). Lean body mass (Fig. 1d) as well as muscle and heart mass (data not shown) were not affected by VWR-induced active lifestyle. Running wheel usage was not different between week 7 and 11, and total daily time spent for VWR was almost 6 hrs (299 ± 12 min in week 7; 298 ± 12 min in week 13). This resulted in a significantly improved endurance exercise capacity which was 2.7 fold increased by VWR by week 15 (Fig. 1e) confirming previous data obtained with a comparable study setup^19,20^. Skeletal muscle citrate synthase (CS) activity as a marker for mitochondria content was not changed by VWR (Fig. 1f). Importantly, plasma creatine kinase (CK) activity as a marker of skeletal muscle damage induced by strenuous exercise^22^ was highly increased 3 hours post exercise in sedentary, but not in active mice (Fig. 1g). At the molecular level, VWR was able to induce skeletal muscle specific adaptations (Fig. 1h). Gene expression of the transcriptional coactivator peroxisome proliferator-activated receptor gamma coactivator 1-alpha (*Pgc1α)*, a key regulator of mitochondrial function and oxidative metabolism, was increased by forced exercise in quadriceps 3 hr post-run in both active and sedentary mice. PGC1α is important for skeletal muscle exercise adaptations such as increased mitochondrial number and function, and induction of a fiber type shift to more oxidative muscle fibers^23,24^ as well as a key regulator of skeletal muscle and systemic lactate homeostasis^25^. Gene expression of activating transcription factor 3 (*Atf3*), a marker of the UPR, was over 20-fold increased immediately after exercise (0 hr post-run) in sedentary mice and further enhanced 3 hr post-run, whereas in active mice only a minor, transient increase could be observed confirming previous findings obtained with forced exercise training^7^. GDF15 is induced by cellular stress in various tissues and released as a myokine under pathophysiological conditions related to mitochondrial myopathies^9^. Here, muscle *Gdf15* gene expression was increased acutely by exercise (0 hr post-run) in sedentary mice only while returning to baseline levels 3 hr post-run (Fig. 1h). It should be noted that GDF15 mRNA levels in skeletal muscle are very low compared to other tissues. Using quantitative real time PCR we found cycle threshold (ct) values in muscle close to 31 in the basal state. Even acutely after exercise (0 hr post-run) GDF15 ct values in sedentary mice were still above 28, while returning to ct values around 30 at 3 hr post-exercise. Importantly, *Gdf15* gene expression pattern in skeletal muscle was not reflected by circulating GDF15 levels which were only marginally affected by exercise. Plasma GDF15 was very similar pre- and post-exercise in sedentary mice and there were no significant differences between active and sedentary mice (Fig. 1i). There was a tendency for reduced GDF15 levels during recovery (3 hr post-run) which is contrary to findings in humans^10^.

### Obesity-induced circulating GDF15 is not affected by active lifestyle mimic

Since GDF15 levels are known to be increased in obesity^13,18^, and VWR improves metabolic features in mice exposed to a high fat diet^26^, we next investigated the effect of long term VWR on exercise capacity, obesity associated metabolic features, and GDF15 expression. To this effect, mice were fed a high fat diet for the induction of obesity. Similar to the first experiment, mice were provided with a running wheel at 7 wks of age (active group) using a sedentary group with no running wheel as a control. Treadmill endurance tests were performed at 7, 15, and 35 wks of age and mice were sacrificed one week after the last treadmill test.

**Figure 2:**
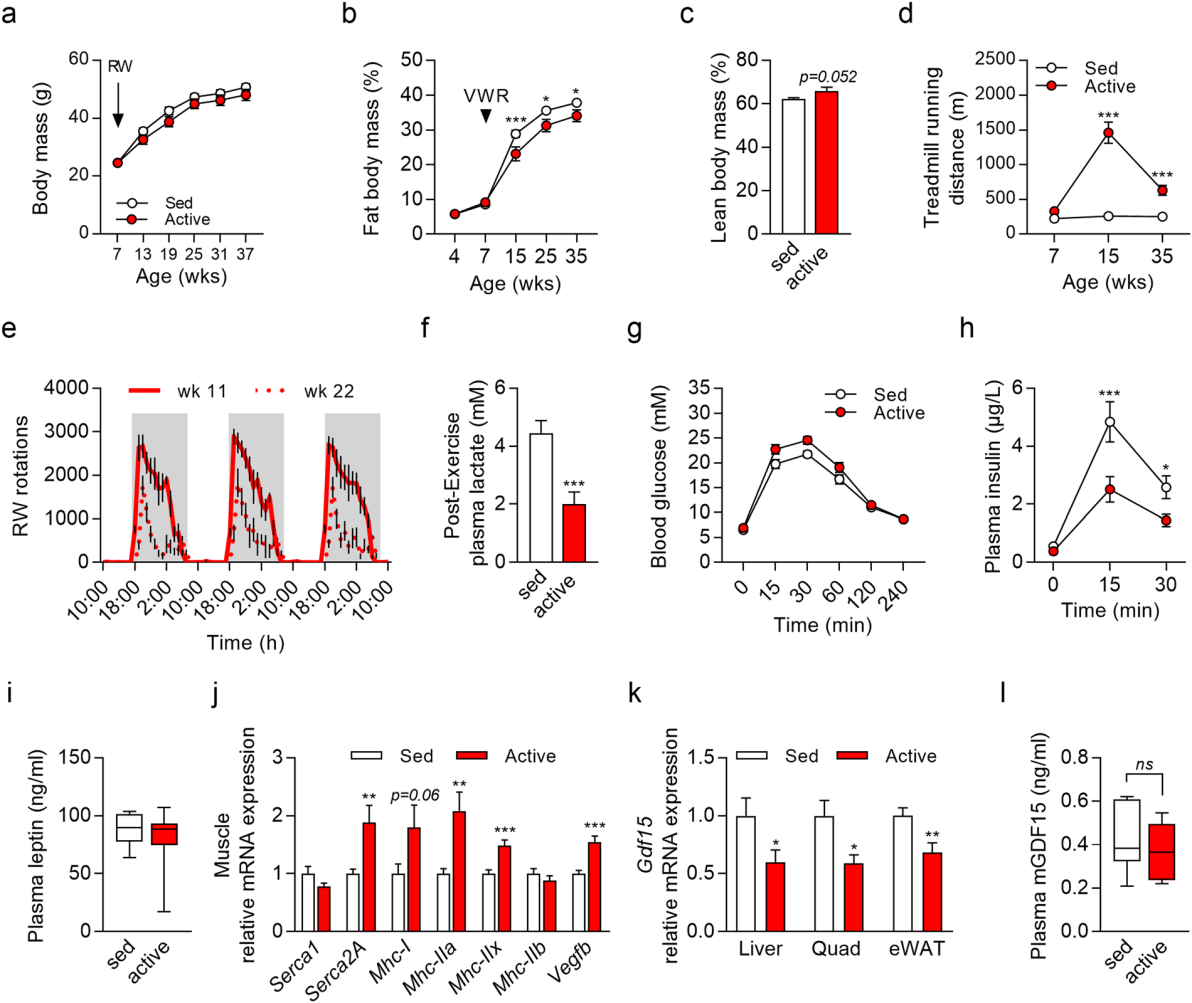
Obesity-induced circulating GDF15 is not affected by voluntary wheel running (VWR) independent of active lifestyle-induced adaptations. Male mice were fed a semisynthetic high fat diet throughout life to induce obesity. Half of the mice were provided a running wheel from 7 wks of age (active group) and all mice were subjected to an exhaustive treadmill test at 7, 15 and 35 wks of age. Animals were sacrificed one week after the last treadmill test. (**a**) Body mass and (**b**) fat mass development (n = 11–12 per group); (**c**) Body lean mass at time of sacrifice; (**d**) Development of exercise capacity determined by forced treadmill exercise until exhaustion (n = 12 per group); (**e**) Usage of running wheel on three consecutive days in week 11 and week 22 of age (n = 8 per group); (**f**) Plasma lactate levels measured immediately after the exhaustive exercise bout at 15 and 35 wks of age (n = 12–13 per group); (**g**) Blood glucose, and (**h**) plasma insulin during an oral glucose tolerance test performed in week 32 (n = 8–12 per group); (**i**) Plasma leptin levels (n = 11 per group); (**j**) Muscle (quadriceps) gene expression and (**k**) *Gdf15* gene expression in liver, quadriceps (Quad) and epididymal white adipose tissue (eWAT) (n = 11 per group); (**l**) Plasma GDF15 levels (n = 11 per group). Data are presented as mean + SEM (**a**–**h**,**j**,**k**) or as box plot with whiskers indicating minimum and maximum values (**i**,**l**). When SEM is not visible it lies within the symbol size. *p < 0.05; **p < 0.01; **p < 0.001 compared to respective sedentary group.

While body weight was not affected by VWR (Fig. 2a) body fat mass was slightly but significantly reduced by VWR (Fig. 2b), and body lean mass increased in tendency (Fig. 2c). Similar to the data obtained in lean mice, exercise endurance capacity (Fig. 2d) was over 5-fold increased by VWR in week 15 compared to sedentary mice while showing an only 2.5 fold but still highly significant increase in week 35 (Fig. 2d). This reduction in exercise capacity is probably due to an age related decline in running wheel usage. It has been reported before that VWR levels showed the most profound drop between 3 and 6 months of age^27^. Figure 2e shows that VWR, which occurred almost exclusively nocturnally, was decreased in week 22 compared to week 11. Daily time spend for VWR was over 4 hours in week 11 (252 ± 7 min) and reduced to under 3 hours in week 22 (160 ± 8 min) and week 32 (170 ± 8 min). However, plasma lactate levels determined immediately after the treadmill endurance test were significantly reduced by VWR at 35 weeks (Fig. 2f) confirming the improved skeletal muscle exercise adaptation^28^. Glucose tolerance was not affected by VWR (Fig. 2g) but insulin sensitivity was apparently increased as evident by lowered plasma insulin levels in response to the glucose load during the oGTT (Fig. 2h). Plasma leptin levels were not affected by VWR (Fig. 2i) despite the slightly decreased fat mass, possibly due to the fact that these mice were still obese. Body fat content of active mice was 34% (38% in sedentary mice) and thus substantially higher than in lean mice from the first experiment which showed body fat contents of 6% and 8% in active and sedentary mice, respectively.

Skeletal muscle gene expression (Fig. 2j) is suggestive of a fiber type shift in the active group towards slow twitch, oxidative type I fibers (gene expression of sarco/endoplasmic reticulum Ca-ATPase *(Serca1*, *Serca2A*) and myosin heavy chain 1 (*Mhc-I*)) and increased metabolic capacity (expression of vascular endothelial growth factor beta (*Vegfb*)). Interestingly, a transcriptional profiling of *Gdf15* expression in liver, quadriceps (Quad) and epididymal white adipose tissue (eWAT) indicated a significant reduction in all three tissues in active mice compared to the sedentary group (Fig. 2k). Nevertheless, this was not reflected by plasma GDF15 levels which were not significantly reduced by VWR (Fig. 2l). Of note, in both active and sedentary obese mice circulating GDF15 was almost 4-fold higher compared to lean mice (Fig. 1i).

### GDF15 ablation does not affect exercise performance but increases skeletal muscle acute exercise stress response

**Figure 3:**
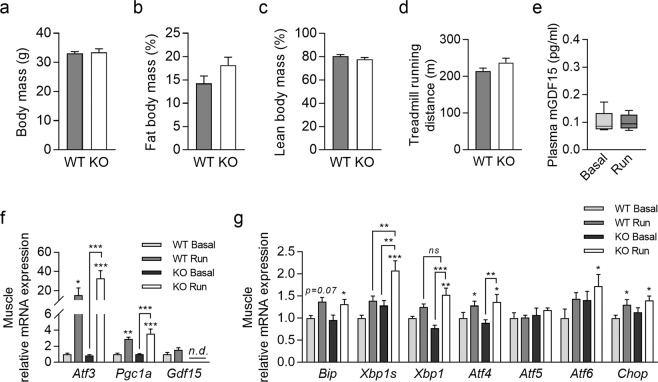
GDF15 ablation has no effect on exercise performance but modulates muscle exercise response. Adult male GDF15-Ko (KO) and wild type (WT) mice were subjected to an exhaustive treadmill exercise test and sacrificed 5 hours (5 hr post-run) after the exercise bout. (**a**) Body mass; (**b**) body fat mass; (**c**) body lean mass; (**d**) exercise capacity (distance run); (**e**) basal and post exercise plasma GDF15 in WT mice; (**f**) skeletal muscle (quadriceps) gene expression of *Atf3*, *Pgc1α* and *Gdf15* and (**g**) skeletal muscle (quadriceps) mRNA levels of UPR marker genes. n = 5–10 per group; data presented as mean + SEM or as box plot with whiskers indicating minimum and maximum values (**e**); *p < 0.05, **p < 0.01, ***p < 0.001 compared to non-exercised control if not otherwise indicated. n.d.: non detectable.

To further characterize the importance of GDF15 for exercise performance and muscle adaptation we investigated exercise capacity in adult male *Gdf15*-ablated (KO) mice. Total body mass and body composition were similar in wildtype (WT) and KO mice (Fig. 3a–c). As shown in Fig. 3d, exercise capacity was not different in KO mice compared to WT suggesting that GDF15 is not necessary for acute exercise response and normal muscle function.

To examine a possible role of GDF15 in the exercise stress response we subjected WT and KO mice to an exhaustive treadmill exercise test and analyzed muscle gene expression 5 hr post- run. This time point was chosen to match to the study of Wu *et al*. who reported for the first time an exercise induced upregulation of the UPR that mediates skeletal muscle adaptation to exercise^7^. As shown in Fig. 3e, circulating GDF15 was not different 5 hr post exercise compared to non-exercised mice, confirming the results obtained with sedentary mice in the first experiment (Fig. 1i). Analogous to this experiment we first examined skeletal muscle gene expression of *Atf3*, *Pgc1a*, and *Gdf15* (Fig. 3f). There was no exercise effect on *Gdf15* expression in WT and *Pgc1a* expression was similarly induced in WT and KO. However, the induction of the UPR marker Atf3 was more pronounced in KO than in WT mice (Fig. 3f) which prompted us to examine in more detail the ER stress and UPR response (Fig. 3g). There were no differences in basal expression of any ER stress and UPR markers. Gene expression of *Bip* (also known as glucose regulating protein 78 (GRP78), an important ER chaperone) and C/EBP homologous protein (*Chop*) was similarly induced post-exercise in WT and KO mice. However, post-exercise mRNA levels of activating transcription factor-6 (*Atf6*), X-box-binding protein 1 (*Xbp1*) and its spliced form (*Xbp1s*) were significantly induced post exercise in KO mice in line with their higher induction of Atf3 (Fig. 3f,g).

## Discussion

Voluntary physical exercise, i.e. an active lifestyle has beneficial effects on health in general as shown in human and rodents^29^. Mechanistic exercise studies in mice are usually performed in sedentary mice confined to a restricted space in their home cages limiting their habitual activity. It can be assumed that these mice are comparable to sedentary humans displaying similar metabolic and skeletal muscle associated features. Here we show that in both lean and obese mice VWR induces skeletal muscle adaptations to an exhaustive exercise bout. Additionally, in accordance with previous studies^30,31^ we found an improvement of glucose homeostasis by VWR in obese mice. These facts together with the blunted lactate increase after exhaustive exercise indicate that VWR induces both metabolic and skeletal muscle specific exercise adaptations. We therefore propose the use of VWR in mice as a tool to study muscle and metabolic adaptation to exercise.

It is well accepted that exercise induces the release of a number of cytokines into the circulation to modulate an adequate metabolic response. One of the best known and characterized examples is the release of IL-6 by the muscle, which has been shown to stimulate hepatic glucose release to compensate the increased glucose uptake by skeletal muscle during exercise^32^. Angiopoietin-like Protein 4 (ANGPTL4), a protein involved in the regulation of plasma triglyceride levels, has been shown to be released from the liver and is controlled by the glucagon-to-insulin ratio as well as by cAMP in humans in response to exercise^33^. Similarly, glucagon-to-insulin ratio has been shown to control liver release of fibroblast growth factor 21 (FGF21), a known endocrine metabolic regulator, during exercise^34^.

During the last years, different human studies have pointed at GDF15 as an exercise-induced cytokine in the circulation. Male athletes participating in an ultramarathon foot race showed a strong increase in plasma GDF15 at the end of the race^35^. In professional rugby players circulating GDF15 was significantly increased after a session of intense training^11^ and an increase in serum GDF15 was also observed in football players of the Spanish Football League 12 h after a match^36^. Recently, GDF15 concentrations were examined during and after a controlled vigorous submaximal exercise in young, healthy males. Plasma GDF15 was gradually increased during exercise reaching maximal levels 3 hr post exercise^10^. Our results in mice suggest that skeletal muscle does not contribute significantly to circulating GDF15 neither during acute nor chronic exercise. Although in sedentary lean mice exhaustive exercise acutely increased skeletal muscle *Gdf15* gene expression, this effect was abolished in active mice. Muscle GDF15 gene expression were not reflected in circulating GDF15, which in lean mice remained at similar levels in both active and sedentary groups before, during, and post exercise. Due to the lack of a reliable anti-GDF15 antibody, we were not able to detect GDF15 protein levels in skeletal muscle by Western blot. Nevertheless, since the mRNA expression level was very low (ct values close to 28) and there was no increase in plasma GDF15 post-exercise, we believe that skeletal muscle does not contribute to circulating GDF15 levels in our experimental setup. In humans, where post exercise increases in circulating GDF15 were observed, evidence suggested that muscle was most probably not its source^10^. Furthermore, in obese mice, VWR reduced *Gdf15* expression not only in skeletal muscle but also in liver and epididymal white adipose tissue. Nevertheless, in obese mice, where GDF15 is known to be endogenously induced by nutritional stress^18^, VWR was unable to rescue GDF15 plasma levels.

Our failure to observe post exercise increases in GDF15 in mice could be due to the effect that our protocol of forced exercise was, although leading to exhaustion, not sufficient to induce training overload and muscle injury. In humans, GDF15 was recently identified as a potential biomarker for the development of the overreaching syndrome, a maladaptive catabolic state caused by endurance training overload^37^. Furthermore, several studies have associated GDF15 expression with tissue injury in the heart^38^, liver^39^, kidney, and lung^40^ among others.

Since GDF15 is considered as a survival or rescue protein^13^ we elaborated the effect of its loss on skeletal muscle exercise stress response. In line with previous findings^41^, GDF15 ablated mice showed no phenotypical differences compared to WT. To the best of our knowledge, effects of GDF15 ablation on exercise capacity have not been studied before. Here we show that endurance exercise capacity and basal mRNA levels of UPR and ER stress markers in skeletal muscle were not affected by GDF15-ablation. However, loss of GDF15 resulted in an amplified post exercise stress response in skeletal muscle, in particular increased mRNA levels of Atf3 and Atf6 and the spliced form of Xbp1 (*Xbp1s*). ATF6 is one of three ER transmembrane sensors mediating the ER-stress induced UPR. Spliced XBP1 is a powerful transcription factor and together with a cleaved fragment of ATF6 it acts in the nucleus to induce UPR target gene expression^8,42^. The UPR plays a pivotal role in skeletal muscle physiology. On one hand it can mediate adaptations to exercise and promote myogenesis (adaptive UPR), but on the other hand it can lead to muscle atrophy and inflammation (maladaptive UPR)^8^. Exercise training leads to a reduced post-exercise activation of the muscle UPR as an adaptive response, and chronic activation of the UPR can lead to cell death^7,8^. This suggests that the aggravated exercise stress response in GDF15-KO mice represents the maladaptive UPR implying a protective action of GDF15 in muscle. A protective role of GDF15 in skeletal muscle has not yet been identified but has been previously described in the myocardium, another muscle type. More specifically, GDF15 protects from cardiac hypertrophy and loss of ventricular performance^38^ and it is required for survival after myocardial infarction in mice acting as an anti-inflammatory cytokine^43^. This is supported by the fact that transgenic mice expressing human NAG-1/GDF15 present a reduced LPS-induced inflammatory response^44^. Thus, given the fact that loss of GDF15 exacervates gene expression of some UPR regulators and this process is tightly linked to inflammation^45^, we hypothesize that GDF15 might contribute to mitigate acute exercise induced inflammation in skeletal muscle. Whether GDF15 acts in a direct, cell autonomous manner or if it plays an indirect, centrally mediated role remains to be elucidated. We could not detect gene expression of *Gfral*, the only known GDF15 receptor, in skeletal muscle (data not shown) confirming previous results of a multi tissue screen that showed its exclusive expression in hind brain^46^. On the other hand, full length GDF15 was also shown to accumulate in the nucleus where it could inhibit different transcriptional pathways^13^, making both scenarios possible. Nevertheless, since we did not see differences in plasma GDF15 levels between sedentary and active mice (Figs. 1i and 3l) as well as before or after exercise (Fig. 1i) we believe it is very unlikely that GFRAL signaling is affected in our experimental setting.

## Conclusion

Here we show that VWR leads to an improved exercise performance and induces skeletal exercise muscle adaptations in mice. Therefore, we suggest its supply in mouse studies as a mimic of an active lifestyle. Furthermore, our data suggest that skeletal muscle does not contribute to circulating GDF15 in response to acute or chronic exercise and does not seem to be important for physiological muscle function in mice. On the other hand, the aggravation of post-exercise stress response in muscle by loss of GDF15 suggests a possible protective role of GDF15 in exercise-induced muscle injury or inflammation.

## Material and Methods

### Experimental design

All animal experiments were performed in accordance with the EU and German regulations for animal experimentation and welfare. They were approved by the ethics committee of the Ministry for environment, health, and consumer protection (State Brandenburg, Germany, Permission no.GZ-23-2347-26-2010, GZ 2347-9-2016). Three separate experiments were performed using male C57Bl/6J mice bred in house that were single caged and kept in a light- and temperature-controlled facility at 22 °C with a 12 h light-12 h dark cycle and food and water ad libitum. The experimental setup of the first two experiments was based on previous studies where we investigated exercise adaptation in the context of maternal programming by high fat diets^19,20,47^. Male mice were fed semisynthetic diets throughout their life and were born from dams that were already fed the same respective diet. In experiment one, mice were fed a low-fat diet (LFD, 10% energy from fat, 23% energy from protein, 67% energy from carbohydrates) and in experiment two a high fat diet (HFD; 40% energy from fat, 23% energy from protein, 37% energy from carbohydrates). Both diets (Research Diet Services) were based on BIOCLAIMS diets^48^. At 7 weeks of age half of the mice from both experiments were provided a running wheel (RW; TSE Systems, Bad Homburg, Germany) as voluntary exercise training (active group). The sedentary group remained without a running wheel. To determine training efficiency and endurance performance, at an age of 7 weeks (before running wheel exposure) a treadmill running exercise test was performed in all mice. This test was repeated in week 9, 11, and 15 (experiment 1) and week 15 and 35 (experiment 2), respectively. In experiment 1, mice were sacrificed in week 15 either before (basal), immediately after (0 hr post-run), or 3 hours after the treadmill running exercise test (3 hr post-run). During the 3 hr recovery, mice had free access to water and food. In experiment 2, blood was obtained from the tail vein for measurement of lactate directly after the exercise test in week 35 and mice were sacrificed one week after the last exercise test. In a third experiment, we used whole-body *Gdf15*-knockout (KO) mice^39^ kindly provided by Dr. Se-Jin Lee (University of Connecticut School of Medicine, Department of Genetics and Genome Sciences) fed a standard chow-diet (Sniff, Soest, Germany) ad libitum. At around 33 weeks of age a treadmill exercise test was performed in KO and wild-type (WT) littermates, and mice were sacrificed 5 hours after the treadmill running exercise bout (5 hr post-run). All mice were sacrificed using isoflurane and cervical dislocation. Blood was taken by cardiac puncture and tissues were removed, weighed and immediately frozen in liquid nitrogen before storage at −80 °C.

### Body composition

Body composition was measured by quantitative nuclear magnetic resonance spectroscopy (NMR; Minispec MQ10 NMR Analysis Bruker, Karlsruhe, Germany) as previously described^49^.

### Training efficiency and endurance exercise performance

Daily wheel running use was recorded using TSE Systems GmbH (Homburg, Germany). Endurance performance (exercise capacity) was determined by forced treadmill exercise until exhaustion using a six-lane treadmill (Columbus Instruments, Columbus, USA) as described previously^19^. Exercise capacity is indicated as the average treadmill running distance (m) until exhaustion.

### Oral glucose tolerance test

In experiment 2 (obese mice) an oral glucose tolerance test (oGTT) was performed in week 32 after 16 h of fasting. After glucose application by gavage (2 mg glucose/g body weight), glucose concentrations were measured in tail vein whole blood by ContourXt glucose sensor (Bayer AG, Leverkusen, Germany) before glucose application and 15, 30, 60, 120, 240, and 360 min after gavage. Plasma insulin was measured at baseline, 15 min and 30 min after glucose administration and determined by Insulin Mouse Ultrasensitive ELISA (Alpco Diagnostics, Salem, USA).

### Biochemical analytics

Muscle citrate synthase activity was measured as previously described^19,50^. Plasma creatine kinase and lactate were measured using an automated analyzer (Cobas Mira S, Hoffmann-La Roche, Basel, Switzerland). GDF15 plasma concentrations were determined by enzyme-linked immunoassays (ELISA) following manufacturer instructions (Mouse/Rat GDF15 Quantikine ELISA Kit, R&D Systems, USA). Leptin plasma concentrations were determined by ELISA following manufacturer instructions (Mouse/Rat Leptin Quantikine ELISA Kit, R&D Systems, USA).

### RNA isolation and gene expression analysis

RNA isolation and analysis of gene expression using quantitative real-time PCR was performed as described previously^51^. Gene expression was calculated as ddCT using ribosomal protein L13A (*Rpl13a*), beta-2 microglobulin (*B2m*), or beta-actin (*Actb*) as reference genes. Values were normalized to the respective control group set as 1.

### Statistical analysis

GraphPad Prism version 7 (Graphpad Software, San Diego, CA, USA) was used for statistical analysis, with Student’s t-test being used to compare the two groups if normally distributed, or One- or two way ANOVA followed by the Bonferroni post hoc test or non-parametric tests where appropriate. Data are expressed as mean with standard error of the mean (SEM) except plasma concentrations which are presented as box plot with whiskers (min to max). Statistical significance was assumed at P < 0.05.

## Data Availability

The datasets generated during and/or analysed during the current study are available from the corresponding author on reasonable request.
